# Editorial: Insights in Sport and Exercise Nutrition: 2021

**DOI:** 10.3389/fspor.2022.937674

**Published:** 2022-06-17

**Authors:** David C. Nieman, Lynn Cialdella-Kam

**Affiliations:** ^1^North Carolina Research Campus, Human Performance Laboratory, Department of Biology, Appalachian State University, Boone, NC, United States; ^2^Warfigther Performance Department, Naval Health Research Center, San Diego, CA, United States

**Keywords:** sports nutrition, precision nutrition, polyphenols, algae, exercise

This Research Topic focused on new insights in sports nutrition, with an emphasis on novel developments, future directions, and challenges. Sports nutrition research and practice have already started down the path of utilizing sophisticated multiomics measurements to formulate nutrition and training guidance more precisely at the individual athlete level (Guest et al., 2019; Sellami et al., 2022).

The article, “*Multiomics Approach to Precision Sports Nutrition: Limits, Challenges, and Possibilities*,” recommended a biology systems-based approach to improve and individualize sports nutrition guidelines for athletes (Nieman). One central challenge to this approach is that athletes vary widely in the way they respond to exercise and nutrition interventions. This metabolic variation is largely unexplained. Precision nutrition uses multiomics tools and methods to sharpen the scientific certainty needed for specific nutrition recommendations for athletes and fitness enthusiasts. A 2-step process was advocated. The first step would involve collecting as much individual-specific, science-based information as possible on the athlete. Although costs are high, multiomics measurements would improve the capacity to capture individual-specific genetic and metabolite responses to training and nutrition regimens. The second step would focus on developing and delivering individualized nutrition guidance combined with periodic monitoring of health and performance outcomes. This sports nutrition precision approach is an emerging science with years of additional studies of large groups needed to ensure accurate, practical, and individualized nutrition guidance at an affordable cost.

Another review, “*New Opportunities to Advance the Field of Sports Nutrition*,” in this Research Topic highlighted technological innovations that have the potential to significantly improve the quality of sports nutrition support (Jonvik et al.). Advances in wearable technologies that facilitate real-time monitoring of athletes in field settings was portrayed as an excellent opportunity to provide more relevant sports nutrition guidelines (Ash et al., 2021). The authors cautioned, however, that too much data and reliance on monitoring devices could be counterproductive. Thus, technological advances should be evaluated in terms of athletic health and performance and be integrated into an entire system of successful support. Along these lines, multiomics-based methods should be adopted once various sports regulatory agencies have had the opportunity to formulate consensus guidelines.

Many of the early studies in sports nutrition focused on hydration and carbohydrate supplementation. Polyphenols have attracted much attention due to their bioactivity and related health benefits, and studies since the mid-1990s have evaluated their utility as a countermeasure to exercise-induced physiological stress. The paper “*Anthocyanin-Rich Supplementation: Emerging Evidence of Strong Potential for Sport and Exercise Nutrition*,” summarized recent studies with dark-colored berries rich in the flavonoid anthocyanins (Copetti et al., 2022; Willems and Blacker). In general, evidence reviewed in this paper supports that acute and chronic intake of anthocyanin-rich fruits before and after prolonged and intense exercise counters exercise-induced inflammation and oxidative stress. Additionally, anthocyanin intake may increase production of nitric oxide during exercise with related effects on vascular function, muscle oxygenation, and performance. The optimal dosing regimen for anthocyanins and other flavonoids is still being investigated, and more sophisticated outcome measures are needed to better understand underlying mechanisms. The metabolic process of anthocyanin absorption, disposition, metabolism, and excretion (ADME) is complex and recent studies utilized untargeted and targeted metabolomics procedures to measure small molecule shifts in athletes following increased intake. Taken together, emerging data support the inclusion of flavonoids along with carbohydrates to mitigate exercise-induced metabolic stress, but more research with better designs is needed (Nieman et al., 2019).

Algae such as spirulina and chlorella are eco-friendly, nutrient rich functional foods that have been touted for their health benefits (Matos et al., 2017; Gouda et al., 2022). Spirulina is high in protein, B-complex vitamins, vitamins E and K, and several minerals including iron, magnesium, and manganese. Tissue culture and animal data support potential metabolic benefits for athletes. However, as emphasized in the mini-review, “*Algae Supplementation for Exercise Performance: Current Perspectives and Future Directions for Spirulina and Chlorella*,” more high-quality research is needed before algae-based supplements are recommended (Gurney and Spendiff). The optimal dosing regimen is still unknown, and few studies have used multiomics-based outcome measures to better understand potential physiological effects.

Sports nutrition research is complex because this area of science attempts to measure how dietary interventions influence the physiological perturbations that occur in response to exercise stress. Metabolic heterogeneity between athletes adds layers of difficulty to this scientific challenge (Cheuvront and Kenefick, 2021). New multiomics tools are now available to better understand these interactive responses at a body systems level for each individual athlete. These methodologies will also improve assessments of the efficacy of novel nutritional interventions such as flavonoid- and algae-based supplements.

## Author's Note

LC-K is a federal employee of the United States government. This work was prepared outside of my official duties. Title 17 U.S.C. 105 provides that “copyright protection under this title is not available for any work of the United States Government.” Title 17 U.S.C. 101 defines a U.S. Government work as work prepared by a military service member or employee of the U.S. Government as part of that person's official duties.

## Author Contributions

DN and LC-K wrote this editorial and agree to be accountable for the content of the work. All authors contributed to the article and approved the submitted version.

## Author Disclaimer

The views expressed in this article reflect the results of research conducted by the author and do not necessarily reflect the official policy or position of the Department of the Navy, Department of Defense, nor the United States Government.

## Conflict of Interest

The authors declare that the research was conducted in the absence of any commercial or financial relationships that could be construed as a potential conflict of interest.

## Publisher's Note

All claims expressed in this article are solely those of the authors and do not necessarily represent those of their affiliated organizations, or those of the publisher, the editors and the reviewers. Any product that may be evaluated in this article, or claim that may be made by its manufacturer, is not guaranteed or endorsed by the publisher.
